# To the Editors of the Medical and Physical Journal

**Published:** 1806-10

**Authors:** 


### *To the Editors of the Medical and Physical Journal.*

GENTLEMEN,

I Beg pardon of your York Correspondent, Practicus,* for having doubted the fidelity of his representation relative to Medical Degrees in the University of Cambridge; but it seemed so extraordinary that the practice of Surgery should disqualify for the practice of Medicine, and the interpretation of the statute "De Studiosis Medicinæ" seemed so wild, that however unjust the Heads might be, I could not suppose the Senate would sanction a measure, which would involve their character, violate their rights, and, as I now find, was made to gratify the private passions of an individual, to whom chance gave a temporary sway in the government of our University.

But, Gentlemen, on a visit to Cambridge, at the Commencement, I find his statement quite correct, and the Heads have usurped a power of interpreting our statutes to any latitude they please, however repugnant to the spirit or letter of them. You must not think, however, that the body of the Senate is in any degree implicated, for the Masters of Arts speak with freedom of the grossness of the interpretation, and of the motives of the Interpretors; as it is notorious, that it was made to exclude an individual,† who, I understand, had been a pupil in the best medical schools in England, and had visited many foreign ones.‡ His rising fame exciting the jealousy of two of the physicians in Cambridge, they resolved to exclude him from medical honours, if they should fail in diminishing his medical emoluments.

The most active person in procuring this Interpretation, and who gave this curious *translation* to queen Elizabeth's statute, was Dr. Davy, formerly an apothecary's apprentice in Norfolk; and surely, if the education which usually falls to the lot of that class of men, is no objection to the attainment of medical honours, the education of a Surgeon offers none.

Your

* Vide Medical Journal, page 554 of the last volume.

† Mr. Thackery. ‡ I find this gentleman kept his Terms, and attended every duty required by his College or expected by the University; yet he received no hint that his previous practice as a Surgeon would disqualify him as an M. D. till the period when, by the custom and constant usage of the University, he might claim an Examination, which was refused him.

Your correspondent seems to throw all the odium on the Professor of Physic; but he merely expressed his doubts: it was Dr. Davy who canvassed the Heads, and by the influence of the office he then held carried *his* interpretation, as personal to the individual* it was meant to exclude, as it is disgraceful to our University to record it on the Statute Book.

If the fountain-head, from whence springs or ought to spring the stream of medical knowledge, is so tainted, and medical honours are conferred where no medical information can be acquired, the necessity of Reform is indeed as obvious as the evil is pressing. The letter of Practicus is short, but it is very full, and deserves the attention of the Legislature; and it is hoped the College of Physicians will not sleep at their post.

*Canterbury, Aug.* 14, 1806. CLERICUS.

* Some of the Heads were so struck with this act of injustice to Mr. Thackery, that seven of them signed a testimonial that he might petition for a mandamus degree with the greatest propriety.

To prove how personal it was to Mr. T. was evinced last year, in the case of Mr. Cope, of St John's College, who was Surgeon in the Mediterranean, and for his meritorious services was made *Inspector of Hospitals*. This was thought sufficient, according to the interpretation, to exclude him from a Medical Degree; but the Vice Chancellor, fearful of offending his College, declared "its duties were the same as a House Visitor at an Hospital. After the interpretation he had made, I was not surprised at the illustration he has given.

